# Home Oral Care with Biomimetic Hydroxyapatite vs. Conventional Fluoridated Toothpaste for the Remineralization and Desensitizing of White Spot Lesions: Randomized Clinical Trial

**DOI:** 10.3390/ijerph19148676

**Published:** 2022-07-16

**Authors:** Andrea Butera, Simone Gallo, Maurizio Pascadopoli, Mona A. Montasser, Mohammad H. Abd El Latief, Gioia Giada Modica, Andrea Scribante

**Affiliations:** 1 Unit of Dental Hygiene, Section of Dentistry, Department of Clinical, Surgical, Diagnostic and Pediatric Sciences, University of Pavia, 27100 Pavia, Italy; andrea.butera@unipv.it (A.B.); gioiagiada.modica01@universitadipavia.it (G.G.M.); 2 Unit of Orthodontics and Pediatric Dentistry, Section of Dentistry, Department of Clinical, Surgical, Diagnostic and Pediatric Sciences, University of Pavia, 27100 Pavia, Italy; 3 Department of Orthodontics, Faculty of Dentistry, Mansoura University, Mansoura 35516, Egypt; mmontasser11@yahoo.com (M.A.M.); mohammadhasan@mans.edu.eg (M.H.A.E.L.)

**Keywords:** white spot lesions, remineralization, fluoride, biomimetic hydroxyapatite, toothpaste

## Abstract

Introduction: Biomimetic hydroxyapatite-based toothpastes have been investigated in recent years for their remineralizing activity on dental surfaces. The aim of the present study was to evaluate the efficacy of toothpaste containing biomimetic hydroxyapatite versus a 1450 pppm fluoride one in promoting the remineralization and desensitization of white spot lesions. Methods: 40 patients were randomly assigned to two different domiciliary oral hygiene treatments: toothpaste containing 1450 ppm of fluoride (control group) and toothpaste containing biomimetic hydroxyapatite (experimental group). Dental sensitivity/pain and dental erosion were assessed at baseline and after 15, 30, and 90 days using the following indexes: Schiff Air Index (SAI), Visual Analogue Scale (VAS), and Basic Erosive Wear Examination (BEWE). Results: Data were submitted for statistical analysis. SAI significantly decreased after 3 months (T3) of treatment only in the Trial group (*p* < 0.05). VAS values significantly decreased at T2 in the trial group (*p* < 0.05) with a further significant reduction at T3 (*p* < 0.05). BEWE scores did not significantly vary during the follow up neither in the trial nor in the control group. Conclusions: The hydroxyapatite-based toothpaste tested caused a reduction of hypersensitivity/pain values higher than conventional fluoride toothpaste.

## 1. Introduction

Enamel demineralization represents an important clinical concern in dentistry. The formation of white spot lesions (WSLs) is the first sign of dental caries, usually appearing as chalky white areas on the tooth surface. The subsurface porosity caused by demineralization gives the lesion a milky appearance that can be found on the smooth surfaces of teeth. This defect of the enamel, besides representing an aesthetic concern, can also be the cause of dental hypersensitivity [1].

The rise of white spot lesions (WSLs) is particularly frequent in patients undergoing orthodontic therapy, with a prevalence ranging from 25% to 46% [2,3,4]. These lesions most commonly occur in the cervical part of the middle third of the crowns of first molars, lateral incisors, and canines [5]. WSLs appear as white opaque lesions after air-drying the teeth. This process derives from a demineralizing process of the enamel. 

Considering its supersaturation with calcium (Ca^2+^) and phosphate (PO_4_^3−^) ions, saliva has a protective role towards enamel with these ions diffusing into deficient lesions thus promoting remineralization [6]. However, this process is not only slow but also insufficient to completely remineralize existing lesions. Therefore, additional agents are required to exert a synergic action [7]. 

According to current literature, fluoride seems to have a high efficacy in both caries prevention and remineralization of initial lesions, but several limitations are related to its use [8,9]. In particular, its efficacy is lowered when oral cavity pH decreases below 4.5; additionally, a proper concentration of Ca^2+^ and PO_4_^3−^ is required for fluoride to be effective [6]. Moreover, only the most superficial layer of the enamel is affected by the remineralizing process, whereas the core of the lesion is not involved [7]. However, the most significant shortcoming associated with fluoride use is the overcoming of the safety doses, exposure to the risk of fluorosis (in case of children), and toxicity [10]. 

Based on the aforementioned considerations, new agents have been required to overcome the limits of fluoride, and one of the most recent technologies is represented by biomimetic hydroxyapatite (HAP) applied in form of microcluster or as nanocrystalline form [11,12]. Biomimetic materials are the result of research efforts to use the tissue engineering technology to shift from enamel remineralization to enamel regeneration [12]. The HAP is chemically similar to the apatite constituting the human enamel crystals and recent research has demonstrated its efficacy for both prevention and remineralization of enamel decays [7,13]. 

Previous research utilizing scanning electron microscope (SEM) and energy-dispersive spectroscopy (EDS) analyses has demonstrated the deposition of biomimetic hydroxyapatite administered through toothpaste for domiciliary use [14]. Additionally, the evaluation of enamel surface roughness has been extensively considered in literature to assess the remineralization promoted by dental products [15].

The present study aimed to compare the efficacy of biomimetic hydroxyapatite concerning fluoride in improving enamel remineralization of WSLs and reducing dental hypersensitivity. The null hypothesis of the study was that no significant differences occur between the two substances considered. 

## 2. Materials and Methods

### 2.1. Study Design 

This was a double-arm parallel, active controlled, randomized clinical trial. The study was conducted from May 2021 until October 2021. The study protocol was approved by the Unit Internal Review Board (approval 2021-0324) and registered on the clinicaltrials.gov (accessed on 1 July 2022) registry (registration number: NCT04908293)

The study was conducted according to the Principles of the Declaration of Helsinki on experimentation involving human subjects. The present trial was conducted following the CONSORT statement. Written informed consent was obtained from all the subjects involved. 

### 2.2. Participants 

Patients’ enrollment, data collection, and statistical analyses were conducted at the Unit of Dental Hygiene, Section of Dentistry, Department of Clinical, Surgical, Diagnostic and Pediatric Sciences, University of Pavia, 27100 Pavia, Italy. 

The inclusion criteria were: age 18–40 years; patients with at least one WSLs on enamel vestibular surface with Score 1 [4]; dental sensitivity positive to air stimulus (Schiff Air Index ≥ 1) on WSLs teeth; regular diet without intolerances; regular salivation; correct oral hygiene habits with domiciliary use of an electric toothbrush; no intake of medications during the three months before the recruitment as well as during the entire follow up; patients that sign the informed consent to participate in the study.

The following exclusion criteria were considered: patients with low compliance or motivation to participate in the study; pregnant or breastfeeding patients; periodontal disease; smoking; recent use of professional and home fluoride products.

### 2.3. Interventions and Outcomes

At the baseline (T0), patients were instructed by a dental hygienist to proper oral hygiene procedures with a manual toothbrush with soft bristles (Toothbrush Sensitive Teeth, Coswell SPA, 40050 Funo di Argelato, Bologna, Italy). After that, they were randomly divided by the same operator into two groups:

- Control group, in which patients had to use Colgate^®^ Protection Caries toothpaste (Colgate-Palmolive, New York, NY, USA) for home oral care twice a day;
- Trial group, in which patients had to use Biorepair^®^ Advanced Sensitive toothpaste (Coswell S.p.A., Funo di Argelato, BO, Italy) for home oral care twice a day.

The compositions of the two types of toothpaste are shown in Table 1.

The following primary outcomes have been assessed by another dental hygienist not involved in the previous phases in order to guarantee blinding: sensitivity and pain assessed by Schiff Air Index (SAI) [16] and Visual Analogue Scale (VAS), [17] and tooth structure loss assessed by Basic Erosive Wear Examination (BEWE) index [18]. In particular, SAI is an index that evaluates the state of dental sensitivity and its values range from 0 to 3; VAS is an index that evaluates pain whose values range from 0 to 10. Finally, BEWE evaluates the state of dental erosion and its values range from 0 to 3 [19]. As shown in Table 2, the clinical parameters were collected at the baseline (T0) and after 15 (T1), 30 (T2), and 90 days (T3) using a manual periodontal probe (UNC probe 15; Hu-Friedy, Chicago, IL, USA).

### 2.4. Sample Size 

Sample size calculation (Alpha 0.05; Power = 95%) for two independent study groups and a continuous primary endpoint was performed concerning the variable “Schiff Air Index”. The following mathematical formula was used for sample size calculation:

$$\mathrm{Sample}\;\mathrm{size}=\frac{Z_{\left(1-\frac{\alpha }{2}\right)}^{2}p\left(1-p\right)}{d^{2}}$$

where $Z_{\left(1-\frac{\alpha }{2}\right)}$ is the standard normal variate corresponding to 1.96 at 5% type 1 error, *p* is the expected proportion in population expressed as decimal and based on previous studies, and finally d is the confidence level decided by the researcher and expressed as decimal too.

An expected value of 0.41 was hypothesized and the expected difference between the means was supposed to be 0.56 with a standard deviation of 0.49 [16], therefore 20 patients per group were required for the study.

### 2.5. Randomization

Using a block randomization table, the data analyst generated a randomization sequence, considering a permuted block of 40 participants. Based on previously prepared sequentially numbered, opaque, sealed envelopes (SNOSE), an operator executed the professional oral hygiene procedures and then assigned teeth to the respective treatment.

### 2.6. Blinding and Reliability

For the domiciliary oral hygiene procedures, the two toothpastes were concealed. Neither the operator nor the patients were aware of the treatment administered. The data analyst was blinded for the allocation. 

Calibration was performed and the reliability of the operator was calculated by repeating BEWE assessment for 10 patients after 1 week, obtaining an agreement of 91%.

### 2.7. Statistical Analysis

Data were submitted to statistical analysis with R Software (R version 3.1.3, R Development Core Team, R Foundation for Statistical Computing, Wien, Austria). For each group and variable, descriptive statistics (mean, standard deviation, minimum, median, maximum) were calculated. SAI was calculated as a pure value; BOP and PI were calculated in percentages. Data normality was assessed with Kolmogorov–Smirnov test. For SAI and VAS variables, inferential comparisons among groups were performed using ANOVA with a post hoc Tukey test. For BEWE variable comparisons were conducted with the Kruskal Wallis test using the Mann Whitney’s U test as post hoc. For inter- and intra-group comparisons, a letter-based comparison has been adopted [20]. Significance was predetermined for *p* < 0.05 for all statistical tests.

## 3. Results

### 3.1. Participants Flow and Baseline Data 

**Figure 1:**
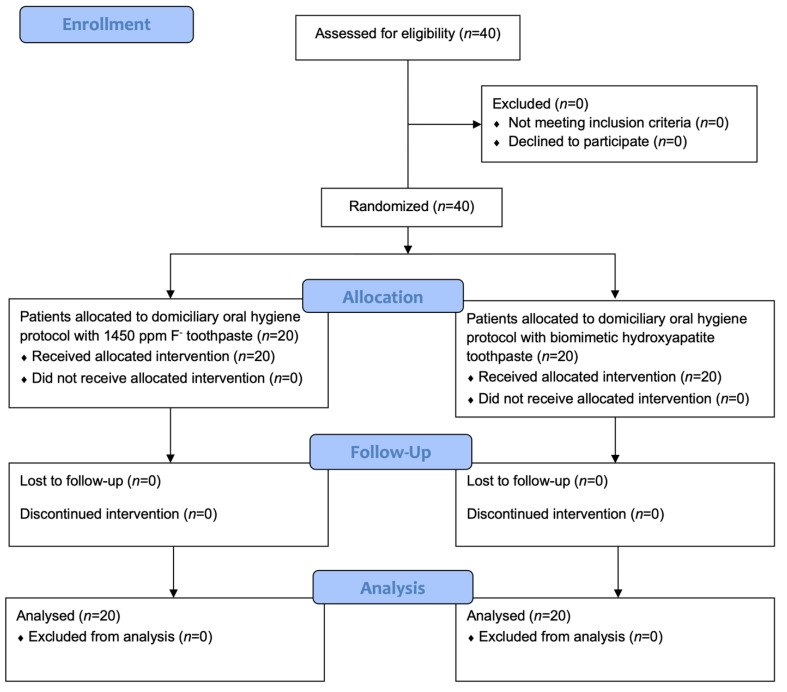
CONSORT flow chart.

Figure 1 shows the flow chart of the study. After screening, 40 patients fulfilling the inclusion criteria were recruited. At the end of the last follow-up (T3), all patients completed the study. Recruitment started in May 2021 and ended in July 2021. The study ended in October 2021. No harms related to any of the two interventions were recorded. 

The demographic characteristics of the study sample are shown in Table 3. 37.5% were female and the overall mean age was 24.9 ± 4.61years (range, 18–37 years) (Table 3). 122 white spots were found in the Control group, in respect to 110 white spots in the Trial group. 

### 3.2. Schiff Air Index (SAI) 

SAI values (Table 4) resulted significantly reduced after 3 months (T3) of treatment only in the Trial group (*p* < 0.05). A decrease was noticed at T2, but it was not significantly different neither for inter- nor for intragroup comparisons (*p* > 0.05). In the Control group, a slight reduction was assessed among the time frames of the study, but with no statistically significant intragroup differences (*p* > 0.05). However, no intergroup significant differences were observed.

### 3.3. Visual Analogue Scale (VAS) 

VAS values for pain (Table 5) exhibited a reduction among the time frames of the study. In the Control group, the reduction was mild and with no intragroup significant differences (*p* > 0.05). In the Trial group, VAS values at T2 resulted significantly reduced from T0 and T1 (*p* < 0.05) and a higher reduction was noticed at T3 (*p* < 0.05). However, no significant intergroup comparisons were found between trial and control groups at the same time intervals (*p* > 0.05).

### 3.4. Basic Erosive Wear Examination (BEWE) 

BEWE values (Table 6) resulted in significant difference between the two groups (*p* < 0.05), but no intragroup changes were assessed among the time frames of the study (*p* > 0.05). 

## 4. Discussion

Dental erosion is a process deriving from the loss of the dental surface because of repeated exposure to acidic agents. In literature, different materials have been tested for their remineralizing effect on both enamel and dentin, among which fluoride-based varnishes, casein phosphopeptide-amorphous calcium phosphate pastes, and biomimetic hydroxyapatite [21,22]. 

The null hypothesis of the study was partially rejected. As regards tooth sensitivity, assessed using SAI, no significant differences were found in the control group from the baseline to the end of the follow-up, whereas in the trial group a significant reduction was assessed at T3 (90 days). These findings agree with a previous randomized clinical trial by Vano and colleagues [23] who compared the efficacy in reducing dentin hypersensitivity of a dentifrice formulation containing nano-hydroxyapatite with a fluoride dentifrice and a placebo. Statistically significant lower values of sensitivity were reported for the group assigned to the hydroxyapatite toothpaste compared to participants assigned to the fluoride toothpaste in the control group, at 2 and 4 weeks respectively. The Authors concluded that nano-hydroxyapatite in fluoride-free toothpaste is effective in desensitizing and providing quick relief from symptoms. These findings were also confirmed by other researchers [24,25]. 

Additionally, in the present study, dental sensitivity was evaluated utilizing a VAS score. These values did not significantly change in the control group during the entire follow-up, whereas in the trial group a significant reduction was assessed at 30 days (T2) with a further significant reduction at 90 days (T3). The VAS score method has been used in previous studies to evaluate changes in dentine hypersensitivity [26,27].

Finally, in the present report, BEWE score was measured. This index was designed to evaluate dental erosion, did not significantly vary neither in the control nor in the trial group at any time point. Accordingly, based on the results obtained here, the hydroxyapatite-based toothpaste tested in this study was not more effective than conventional fluoride toothpaste in reducing erosive wear, but it was able to reduce dental hypersensitivity/pain assessed after at least a 30-day-use. 

Previous research aimed to assess the in vitro visual efficacy of a biomimetic nano-hydroxyapatite remineralizing solution (Biorepair^®^ Repair Shock Treatment) in a hypomineralized enamel surface, as well as its effect on enamel microhardness [28]. The application of the remineralizing solution induced a significant in vitro reduction of the demineralized areas after 7 days of application, which agrees with our findings. Additionally, remineralized enamel showed lower microhardness values than intact enamel but significantly higher than demineralized one. However, in vitro results cannot be directly compared to those obtained under real in vivo conditions.

The specific action of biomimetic hydroxyapatite has been studied using morphological and chemical analyses [14]. In particular, SEM morphologic evaluation has shown that the hydroxyapatite-based toothpaste caused in vivo a marked deposition of the mineral on polymeric frameworks. Additionally, EDS quantitative analysis confirmed that the product significantly increased calcium, phosphorus, and silicon deposition. 

The results obtained in the present study confirm those obtained in previous similar randomized clinical trials. For instance, Badiee and colleagues randomized 50 patients, immediately after orthodontic debonding, to the use of a nano-HA containing vs. fluoride containing toothpaste [29]. Examinations were conducted at 1, 3, and 6 months’ intervals. According to the results of the study, the nano-HA containing toothpaste tested performed better than fluoride containing, both in terms of the amount of the remineralization and decrease of the lesion extent. 

Lelli and colleagues compared the effect of fluoride and hydroxyapatite based toothpasted on teeth extracted for orthodontic purposes: after randomizing patients to the use of the respective product for eight weeks, teeth extractions were conducted [30]. By means of SEM, X-Ray diffraction, ad Infrared analyses, the use of a Zn-CHA nanocrystals toothpaste was shown to lead to a remineralization/repair of the enamel surface, by deposition of a hydroxyapatite-rich coating, whereas the use of the fluoride toothpaste did not appreciably change the enamel surface. Interestingly, this “ex vivo” protocol allows a direct transfer of the laboratory results to the clinical setting. In fact, as regards in vitro reports, a direct comparison with clinical evaluations is not possible due to the influence of many factors under *in vivo* conditions that are not addressed in laboratory studies. 

Whereas in the control group BEWE score was constant throughout the entire follow-up, a slight reduction appeared in the case of using the hydroxyapatite-based toothpaste, despite without a statistical significance. On the opposite, as regards dental hypersensitivity/pain, a significant reduction was assessed only for the group assigned to the hydroxyapatite toothpaste, whereas no alterations were found in the case of the fluoride toothpaste use. In particular, SAI scores were significantly lower in the experimental group after 90 days from baseline, whereas VAS scores were significantly lower even before, precisely after 30 days of use. Based on the previous considerations, although no macroscopic remineralization was found due to the absence of significant alterations of BEWE scores, it can be assumed that the crystals of hydroxyapatite were partially effective in covering dentinal tubules, thus contributing at least to the reduction of VAS and SAI scores. Accordingly, the use for 3 months of the experimental product might have caused the deposition of hydroxyapatite crystals which was effective in reducing dental hypersensitivity but not in contributing to the visual macroscopic remineralization of the treated surfaces. 

The present research covers a hot-topic in dentistry. The comparison between fluoride and hydroxyapatite-based toothpastes is nowadays very debated among both Authors and dentists/dental hygienists. Accordingly, the results of our study contribute to confirm the major efficacy of the latter, thus suggesting to dental practitioners to rely on them.

The main limitation of the present study is related to the fact that pain and hypersensitivity are not objective parameters and therefore could be prone to bias [31]. Moreover, the remineralizing effect has been visually assessed by the operator and the 90 days follow up could result to be relatively short especially for the evaluation of the state of dental erosion by means of the BEWE index. Future studies are required encompassing morphological and chemical evaluations, like SEM and EDS analysis, to analyze the ultrastructure of the surfaces treated with the substances tested. It could be also interesting to perform split-mouth design studies and to compare the efficacy of hydroxyapatite with other remineralizing agents, like casein phosphopeptides-amorphous calcium phosphate (CPP-ACP), as well as to consider the possible combination and interactions of various molecules.

## 5. Conclusions

Despite the limitations mostly related to the subjectivity of the parameters assessed, the present study shows that the use of a toothpaste containing hydroxyapatite could be proposed as a reliable device for the domiciliary management of WSLs because of its efficacy in reducing hypersensitivity more effectively than conventional fluoride toothpaste. 

## Figures and Tables

**Table 1:** Compositions of the materials used in the study.

Toothpaste	Manufacturer	Composition
Biorepair^®^ Advanced Sensitive	Coswell S.p.A., Funo di Argelato, BO, Italy	Aqua, Zinc Hydroxyapatite (18% *w*/*v*), Glycerin, Sorbitol, PEG-32, Xylitol, Cellulose Gum, Silica, Sodium Myristoyl Sarcosinate, Sodium Methyl Cocoyl Taurate, Aroma, Zinc PCA, Cetylpyridinium Chloride, Citric Acid, Sodium Benzoate, Benzyl Alcohol, Phenoxyethanol, Sodium Saccharin, Limonene.
Colgate^®^ Protection Caries	Colgate-Palmolive, New York, NY, USA	Dicalcium Phosphate Dihydrate, Aqua, Glycerin, Sodium Lauryl Sulfate, Cellulose Gum, Aroma, Tetrasodium, Pyrophosphate, Sodium Saccharin, Sodium Fluoride (1450 ppm F⁻), Sodium monofluorophosphate (1000 ppm F⁻).

**Table 2:** Protocol of the study.

Appointment	Procedures
Baseline (T_0_)	Signature to the informed consent for the study (SAI, VAS, BEWE) Assessment of periodontal clinical indexes Professional supragingival and subgingival oral hygiene with piezoelectric and Gracey curettes Periodontal pockets decontamination with Air-flow Plus Motivation to oral hygiene and instruction for the domiciliary treatment: Group 1: toothpaste Colgate^®^ Protection Caries toothpaste (Colgate-Palmolive, New York, NY, USA) Group 2: toothpaste Biorepair^®^ Advanced Sensitive toothpaste (Coswell S.p.A., Funo di Argelato, BO, Italy)
After 15 days (T_1_) After 30 days (T_2_) After 90 days (T_3_)	Reassessment of periodontal clinical indexes (SAI, VAS, BEWE) Professional supragingival and subgingival oral hygiene with piezoelectric and Gracey curettes Periodontal pockets decontamination with Air-flow Plus Further motivation to oral hygiene and continuation of the domiciliary treatment assigned

**Table 3:** Baseline demographic characteristics.

	Control Group (*n* = 20)	Trial Group (*n* = 20)	Total
Age (years)			
Mean (SD)	26.2 ± 5.07	23.6 ± 3.78	24.9 ± 4.61
Min-Max	18–37	18–30	18–37
Sex			
Female	10 (50%)	5 (25)	15 (37.5%)
Male	10 (50%)	15 (75%)	25 (62.5%)
Whitespots (*n*)			
	122	110	232

**Table 4:** SAI scores.

Group	Time	Mean	SD	Min	Median	Max	Significance *
Control	T0	1.65	0.59	1.00	2.00	3.00	A
	T1	1.60	0.60	1.00	2.00	3.00	A
	T2	1.55	0.60	1.00	1.50	3.00	A
	T3	1.45	0.51	1.00	1.00	2.00	A
Trial	T0	1.75	0.72	1.00	2.00	3.00	A
	T1	1.70	0.73	1.00	2.00	3.00	A
	T2	1.15	0.49	0.00	1.00	2.00	A
	T3	0.40	0.50	0.00	0.00	1.00	B

* Means with the same letters are not significantly different (*p* > 0.05).

**Table 5:** VAS scores.

Group	Time	Mean	SD	Min	Median	Max	Significance *
Control	T0	6.15	1.53	2.00	6.00	8.00	A
	T1	5.85	1.69	2.00	6.00	8.00	A
	T2	5.40	1.57	2.00	5.00	8.00	A, B
	T3	5.10	1.55	3.00	5.00	8.00	A, C
Trial	T0	6.80	1.47	3.00	7.00	9.00	A
	T1	6.65	1.50	3.00	6.50	9.00	A
	T2	5.30	1.22	3.00	5.00	8.00	B
	T3	3.60	1.57	0.00	4.00	6.00	C

* Means with the same letters are not significantly different (*p* > 0.05).

**Table 6:** BEWE scores.

Group	Time	Mean	SD	Min	Median	Max	Significance *
Control	T0	2.10	0.72	1.00	2.00	3.00	A
	T1	2.10	0.72	1.00	2.00	3.00	A
	T2	2.10	0.72	1.00	2.00	3.00	A
	T3	2.10	0.72	1.00	2.00	3.00	A
Trial	T0	2.65	0.49	2.00	3.00	3.00	A
	T1	2.65	0.49	2.00	3.00	3.00	A
	T2	2.60	0.50	2.00	3.00	3.00	A
	T3	2.60	0.50	2.00	3.00	3.00	A

* Means with the same letters are not significantly different (*p* > 0.05).

## Data Availability

Data are available upon reasonable request to the corresponding authors.
